# The mitochondrial thiamine pyrophosphate transporter TptA promotes adaptation to low iron conditions and virulence in fungal pathogen *Aspergillus fumigatus*

**DOI:** 10.1080/21505594.2019.1596505

**Published:** 2019-03-28

**Authors:** Jingjing Huang, Zhihua Ma, Guowei Zhong, Donald C. Sheppard, Ling Lu, Shizhu Zhang

**Affiliations:** a Jiangsu Key Laboratory for Microbes and Functional Genomics, Jiangsu Engineering and Technology Research Center for Microbiology, College of Life Sciences, Nanjing Normal University, Nanjing, China; b Department of Hygiene Analysis and Detection, School of Public Health, Nanjing Medical University, Nanjing, China; c Departments of Medicine, Microbiology & Immunology, McGill University, Montréal, QC, Canada; d Infectious Diseases and Immunity in Global Health Program, Research Institute of the McGill University Health Centre, Montreal, QC, Canada

**Keywords:** Thiamine pyrophosphate transporter, low iron adaptation, *Aspergillus fumigatus*, virulence, HapX

## Abstract

*Aspergillus fumigatus* is the most prevalent airborne fungal pathogen that causes invasive fungal infections in immunosuppressed individuals. Adaptation to iron limited conditions is crucial for *A. fumigatus* virulence. To identify novel genes that play roles in adaptation to low iron conditions we performed an insertional mutagenesis screen in *A. fumigatus*. Using this approach, we identified the *tptA* gene in *A. fumigatus*, which shares homology with the *Saccharomyces cerevisiae* thiamine pyrophosphate (ThPP) transporter encoding gene *tpc1*. Heterologous expression of *tpc1* in the *tptA* deletion mutant completely restored the ThPP auxotrophy phenotype, suggesting that Tpc1 and TptA are functional orthologues. Importantly, TptA was required for adaptation to low iron conditions in *A. fumigatus*. The *ΔtptA* mutant had decreased resistance to the iron chelator bathophenanthroline disulfonate (BPS) with severe growth defects. Moreover, loss of *tptA* decreased the expression of *hapX*, which is a major transcription factor indispensable for adaptation to iron starvation in *A. fumigatus*. Overexpression of *hapX* in the *ΔtptA* strain greatly rescued the growth defect and siderophore production by *A. fumigatus* in iron-depleted conditions. Mutagenesis experiments demonstrated that the conserved residues related to ThPP uptake in TptA were also required for low iron adaptation. Furthermore, TptA-mediated adaptation to low iron conditions was found to be dependent on carbon sources. Finally, loss of *tptA* resulted in the attenuation of virulence in a murine model of aspergillosis. Taken together, this study demonstrated that the mitochondrial ThPP transporter TptA promotes low iron adaptation and virulence in *A. fumigatus*.

## Introduction

The unique chemical properties of iron underlie its broad utility as a cofactor for essential cellular processes. Although iron is highly abundant in the earth’s crust, the bioavailability of iron is very low due to its oxidation into sparingly soluble ferric (Fe^3+^) hydroxides by atmospheric oxygen (below 10^−9^ M at neutral pH) [1,2]. Control over access to iron is one of the central battlefields during infection, as pathogens typically face low iron levels in the host (~10^−24^ M free Fe^3+^ in bloodstream) [3–6]. However, excess iron has the potential to catalyze the formation of cell-damaging reactive oxygen species [7]. Thus, virtually all organisms, especially pathogens have evolved precise mechanisms to adapt to both low and high iron conditions.

*Aspergillus fumigatus* is an ubiquitous airborne fungal pathogen of humans [8]. The most severe form of *A. fumigatus* infection, invasive aspergillosis (IA), occurs when inhaled *A. fumigatus* spores germinate into hyphae and invade the lung tissue of immuno-compromised patients [9]. Despite antifungal treatment with currently available antifungal agents, invasive aspergillosis (IA) mortality remains between 50 and 95% [10–12]. *A. fumigatus* primarily employs two high affinity iron uptake systems, reductive iron assimilation (RIA) and siderophores (low molecular mass, ferric iron chelators) to survive in low iron environments [2]. Blocking of RIA through deletion of the *ftrA* gene, which encodes an iron permease, does not affect *A. fumigatus* virulence [13]. In contrast, *A. fumigatus* SidA, which catalyzes the first committed step of hydroxamate siderophore biosynthesis, is essential for virulence, suggesting that siderophore-mediated iron uptake plays a major functional role during fungal infection [13].

Iron homeostasis in *Aspergillus* is regulated by two central transcription factors HapX and SreA. Under low iron conditions, HapX activates siderophore-mediated iron acquisition and represses iron-consuming pathways to conserve iron [14]. During iron-replete conditions, SreA represses iron uptake via the downregulation of siderophore-mediated and reductive iron assimilation to avoid its toxic effects [15]. These two transcription factors are interconnected in a negative transcriptional feed-back loop. Deficiency of HapX, but not SreA, attenuates *A. fumigatus* virulence in murine models of aspergillosis [14,15], emphasizing the crucial role of adaptation to iron limitation in pathogenicity.

To identify novel genes crucial for growth under low iron conditions, we carried out a large-scale *Agrobacterium*-mediated transformation study in *A. fumigatus*. Transformants exhibiting specific defects under iron-limited conditions were selected for further study. Here, we present the identification and functional characterization of a novel low iron adaptation related gene, *tptA*, a homolog of the *Saccharomyces cerevisiae* thiamine pyrophosphate (ThPP) transporter encoding gene *tpc1*. In *S. cerevisiae*, Tpc1 mediates mitochondrial uptake of the essential cofactor ThPP, which is required for the activity of acetolactate synthase (ALS), pyruvate dehydrogenase (PDH) and oxoglutarate dehydrogenase (OGDH) [16]. The study revealed that TptA is necessary for mitochondrial uptake of ThPP and the mitochondrial ThPP import is particularly important in adaptation to iron starvation and virulence in *A. fumigatus*.

## Results

### Identification of the T-DNA-tagged gene *tptA* of *A. fumigatus*

To identify novel genes involved in adaptation to low iron conditions, a T-DNA insertional mutagenesis library containing 5,000 *A. fumigatus* transformants was constructed. Transformants were screened for their ability to grow and/or conidiate on minimal media (MM) in the presence of the iron chelator bathophenanthroline disulfonate (BPS). Seven mutants impaired by low iron conditions were identified. The T-DNA flanking sequences in four of the mutants were cloned successfully using Thermal-asymmetric interlaced (TAIL)-PCR in the mutant genomes [17]. Among these cloned sequences, *sidC* (AFUA_1G17200), *sidF* (AFUA_3G03400) and *sidI* (AFUA_1G17190) were previously reported to be involved in low iron adaptation through mediating siderophore biosynthesis [18,19].

**Figure 1.**
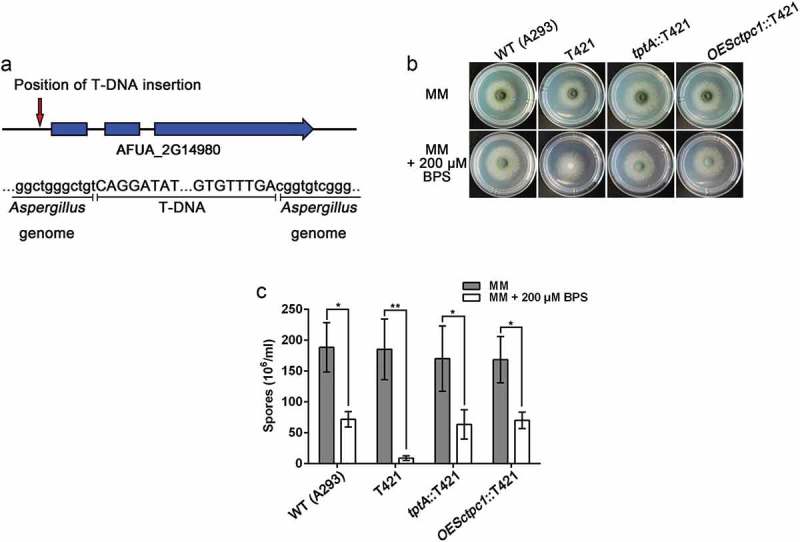
Identification of the T-DNA-tagged gene *tptA* in *A. fumigatus*. (a) Schematic of the T-DNA insertion site in the genome of *A. fumigatus* A293. (b) Colony morphology comparison for the indicated strains grown on solid MM in the presence or absence of 200 μM BPS at 37°C for 48 h. (c) Quantitative total conidial production for the strains shown in panel (b). Data are presented as the means ± SD from three independent replicates. “*” or “**” represents significant differences at *P* < 0.05 and *P* < 0.01, respectively, according to t-test.

In this study, a single mutant T421, which displayed reduced conidiation in the presence of BPS, but normal growth on MM, was selected for further characterization. TAIL-PCR analysis of this mutant demonstrated the T-DNA insertion was located 55 bp upstream of the translational start site of the AFUA_2G14980 gene on chromosome 2 (Figure 1(a)). We named the T-DNA tagged gene *tptA* (thiamine pyrophosphate transporter), as it encodes a predicted protein that showed 28.61% amino acid identity with *S. cerevisiae* Tpc1, a mitochondrial transporter of ThPP [16]. Complementation of T421 with the wild-type *A. fumigatus tptA* allele restored the conidiation defect in the presence of BPS suggesting that dysfunction of *tptA* is responsible for the growth defect in this strain under low iron conditions (Figure 1(b) and (c)).

### TptA is required for growth under low thiamine conditions

**Figure 2.**
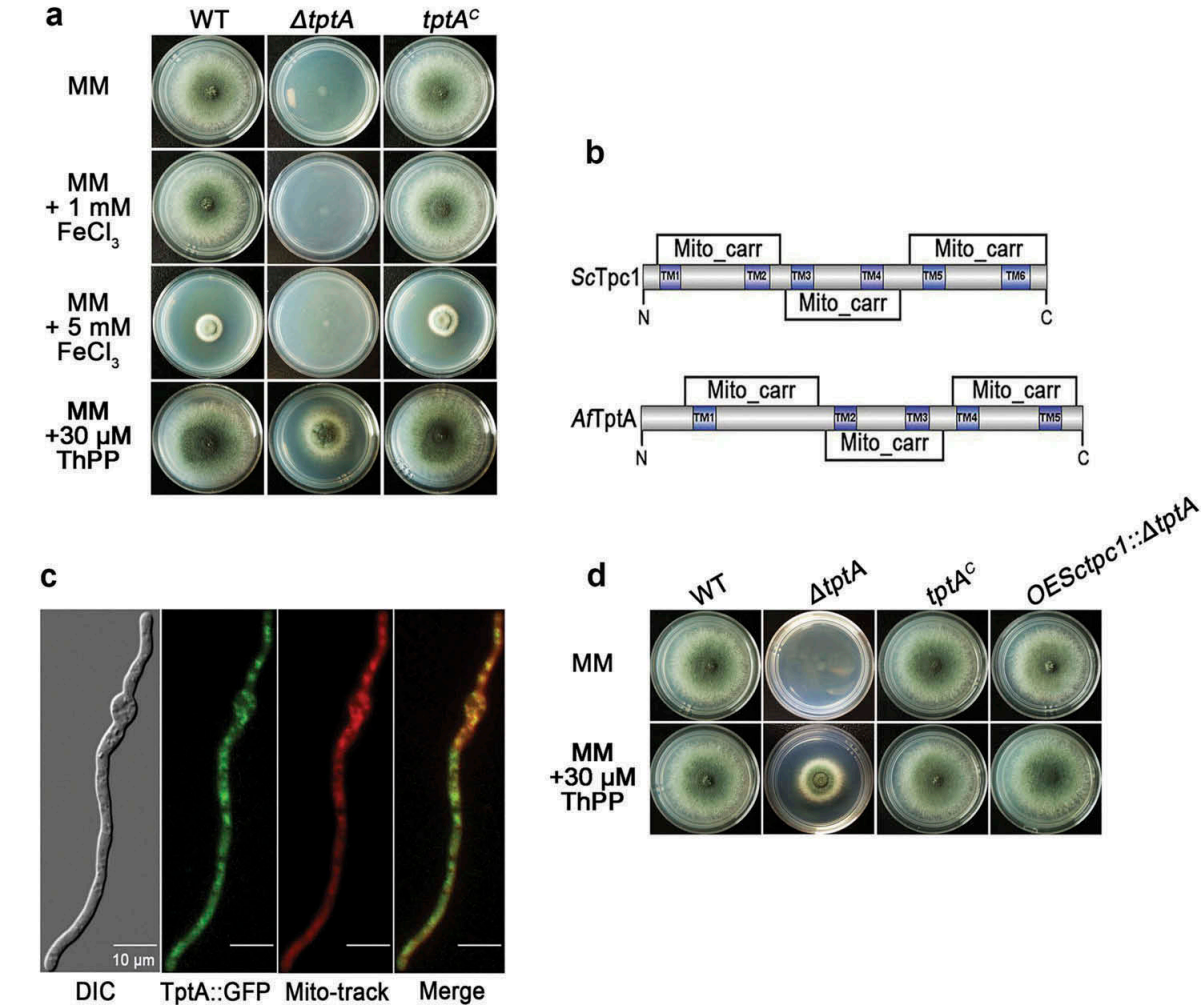
TptA is required for growth under low thiamine pyrophosphate conditions. (a) Comparison of colony morphology for the indicated strains grown on solid MM in the presence of 1, 5 mM FeCl_3_ or 30 μM ThPP at 37°C for 48 h. (b) Comparison of mitochondrial carriers and transmembrane domains between *S. cerevisiae* Tpc1 and *A. fumigatus* TptA. (c) GFP-tagged TptA was located in mitochondria. Mito-tracker was used to visualize mitochondria. Scale bar = 10 μM. (d) Heterologous expression of *tpc1* in the *tptA* deletion mutant completely restored growth of the *ΔtptA* mutant on MM. The indicated strains were grown on solid MM in the presence or absence of 30 μM ThPP at 37°C for 48 h.

To explore the function of *tptA*, a *ΔtptA* mutant was constructed in the A1160 background strain (*Δku80, pyrG1*). The resulting *ΔtptA* mutant was confirmed by PCR and Southern blotting (Figure S1(a–c)). In contrast to the T421 mutant, complete deletion of *tptA* resulted in a mutant that did not grow on MM, even in the presence of 1 or 5 mM FeCl_3_ (Figure 2(a)). The different growth phenotype observed on MM between the *ΔtptA* and T421 mutants is most likely due to partial function of TptA in strain T421 (the T-DNA insertion was in the 5ʹ untranslated region of *tptA vs* complete gene deletion in the *ΔtptA* mutant). Since TptA has been reported to play a role in ThPP transport in *S. cerevisiae* [16], we hypothesized that loss of *tptA* resulted in ThPP auxotrophy. As expected, the addition of ThPP (>10 μM) or thiamine (> 1 μM) restored growth of the *tptA* deletion mutant on MM (Figure S2(a)). The addition of other cofactors such as pyridoxine, riboflavin or inositol did not improve growth of the *ΔtptA* mutant on MM (Figure S2(b)). In addition, it has been reported that the simultaneous addition of valine and isoleucine fully restored the growth of a *Δtpc1* mutant when grow with glucose as a carbon source in *S. cerevisiae* [16]. In contrast, simultaneous addition of all three branched chain amino acids (valine, isoleucine, and leucine) did not restore the growth of the *ΔtptA* mutant (Figure S2(b)).

The mitochondrial carrier family is a large group of structurally-related membrane proteins. Most mitochondrial carriers, including Tpc1 from *S. cerevisiae*, contain three tandem repeat homologous domains of approximately 100 amino acids, and are predicted to have six transmembrane helices (TM1-TM6) with N- and C-termini located in the intermembrane space [20,21]. A SMART protein search predicted a similar secondary structure and topology to other mitochondrial carriers for TptA, except for the presence of five predicted transmembrane helices (TM1-TM5) (Figure 2(b)). To verify the subcellular localization of TptA, the C-terminus of TptA was labelled with GFP and expressed under the control of its native promoter. The phenotype of the TptA::GFP mutant strain was similar to that of the parental wild-type *A. fumigatus* under all tested conditions, indicating that TptA::GFP is fully functional (data not shown). As expected, the TptA::GFP fusion protein co-localized with the mitochondrial marker MitoTracker Red, suggesting that TptA is located within mitochondria (Figure 2(c)). To confirm that *tptA* is a functional ortholog of *S. cerevisiae tpc1, tpc1* was expressed in the *ΔtptA* and T421 mutants, under the control of the constitutive *Aspergillus nidulans gpdA* promoter. As expected, expression of *tpc1* in the *ΔtptA* mutant fully restored ThPP auxotrophy in the *ΔtptA* mutant (Figure 2(d)). Expression of *tpc1* in the T421 mutant restored the conidiation defect in the presence of BPS (Figure 1(c)). Collectively, the cross-species complementation indicates that Tpc1 can fulfill similar cellular functions to TptA in *A. fumigatus.*

### TptA is required for adaptation to low iron conditions

**Figure 3.**
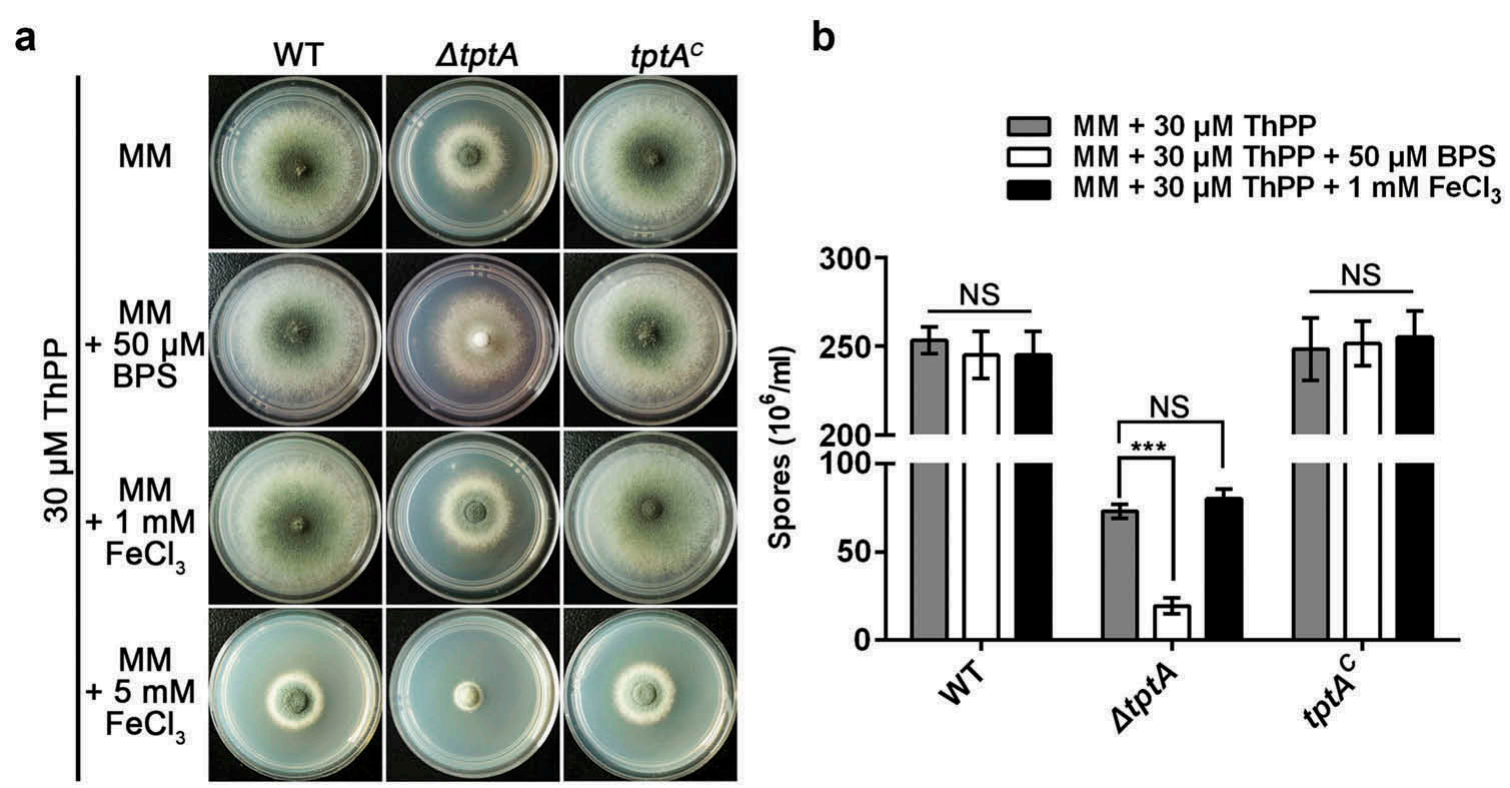
TptA is required for adaptation to low iron conditions. (a) Comparison of colony morphologies for the indicated strains grown on MM media supplemented with 1, 5 mM FeCl_3_ or 50 μM BPS in the presence of 30 μM ThPP. The strains were grown at 37°C for 48 h. (b) Quantitative total conidial production for the strains shown in panel (a). Data are presented as the means ± SD of three independent replicates. “***” represents significant differences at *P* < 0.001 according to t-test. NS, no significant difference.

To characterize the role of TptA in iron homeostasis, growth of the *ΔtptA* mutant was tested on MM containing various levels of iron. To support growth of the *ΔtptA* mutant on MM, 30 μM ThPP was added to MM in the following tests. The *ΔtptA* mutant displayed a consistent BPS-sensitive phenotype with dramatically decreased conidiation, as seen in the T421 mutant (Figure 3(a) and (b)). The addition of 1 mM FeCl_3_ plus 30 μM ThPP did not further improve the growth of the *ΔtptA* mutant compared to ThPP alone. In addition, the *ΔtptA* mutant displayed comparable susceptibility to iron toxicity to the wild-type *A. fumigatus* on MM plus 5 mM FeCl_3_ (iron excess condition) in the presence of ThPP. The *tptA* complemented strain *tptA*^c^ had an identical phenotype to the wild-type *A. fumigatus* strain under all tested conditions, suggesting that the phenotypes exhibited by the *ΔtptA* mutant were caused only by the loss of *tptA* (Figure 3(a) and (b)). Although *S. cerevisiae* Tpc1 has been observed to mediate ThPP uptake, no role for this protein in iron homeostasis has been reported. In this study, we have shown that *A. fumigatus* TptA is involved not only in ThPP transport but also in low iron adaptation.

### Overexpression of *hapX* partly restores the defects of the *tptA* deficient strain in low iron conditions

**Figure 4.**
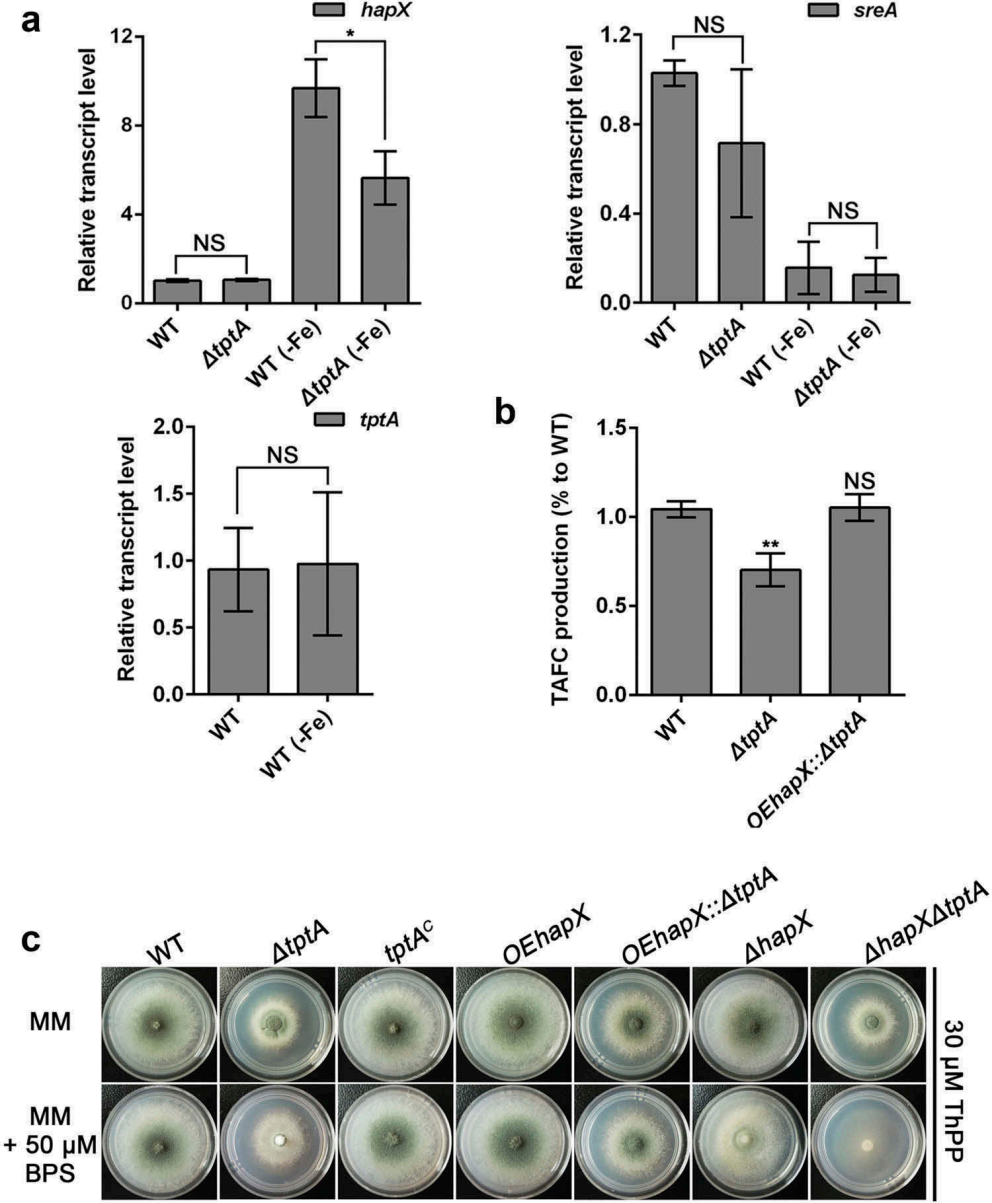
Overexpression of *hapX* partly restores the conidiation defect of the *tptA* deficient strain in low iron conditions. (a) Relative RNA expression of *hapX, sreA* and *tptA* in the indicated strains cultured in MM or MM-Fe broth in the presence of 30 μM ThPP. (b) Compared to WT, lack of *tptA* results in decreased production of TAFC. TAFC was quantified by reversed-phase HPLC analysis from supernatants of cultures grown in MM-Fe broth with 30 μM ThPP for 24 h. TAFC production was normalized to biomass and WT production. (c) Colony morphology comparison for the indicated strains grown on MM media with or without 50 μM BPS in the presence of 30 μM ThPP at 37°C for 48 h. Data are presented as the means ± SD from three independent replicates. “*” or “**” represent significant differences at *P *< 0.05 and *P *< 0.01, respectively, according to t-test.

To probe the mechanism by which loss of *tptA* affects fungal adaptation to low-iron conditions, we tested whether expression of the major iron homeostasis transcription factors, *sreA* and *hapX*, were affected by the loss of *tptA*. Consistent with previous report, the *hapX* transcript level was up-regulated under low iron conditions [14]. However, under these conditions induction of *hapX* in the *ΔtptA* mutant was significantly reduced compared to wild-type *A. fumigatus* (Figure 4(a)). In contrast, there was no difference in the mRNA level of *sreA* between the *ΔtptA* mutant and wild-type *A. fumigatus*. Transcript levels of *tptA* were unaffected by the iron content of the medium (Figure 4(a)).

In agreement with the important role of HapX in mediating the production of the secreted low molecular weight ferric (Fe^3+^) iron siderophore triacetylfusarinine C (TAFC), production of TAFC by *ΔtptA* mutant was only 70% of the WT (Figure 4(b)). To test if reduced *hapX* expression was responsible for decreased TAFC production and the subsequent BPS-sensitivity of the *ΔtptA* mutant, a mutant strain was constructed in which *hapX* was overexpressed in the *ΔtptA* mutant background. As predicted, overexpression of *hapX* in the *ΔtptA* mutant (strain *OEhapX::ΔtptA*) completely restored the TAFC production defect in the *ΔtptA* mutant (Figure 4(b)). Conidiation in the *OEhapX::ΔtptA* mutant was also dramatically increased compared to the *ΔtptA* mutant on both MM and MM plus 50 μM BPS. However, the *ΔhapXΔtptA* mutant had an increased conidiation defect compared to the parental single knockout mutants under iron starvation conditions (Figure 4(c) and Figure S3(a)). In comparison, no significant restoration of biomass was seen in the *OEhapX::ΔtptA* mutant on both MM and in MM-Fe broth (Figure S3(b)). Collectively, the above results indicate that mitochondrial ThPP level affects the expression of *hapX* and the subsequent TAFC production under low iron conditions.

### Conserved residues related to thiamine pyrophosphate transport in TptA are required for adaptation to iron-depleted conditions

**Figure 5.**
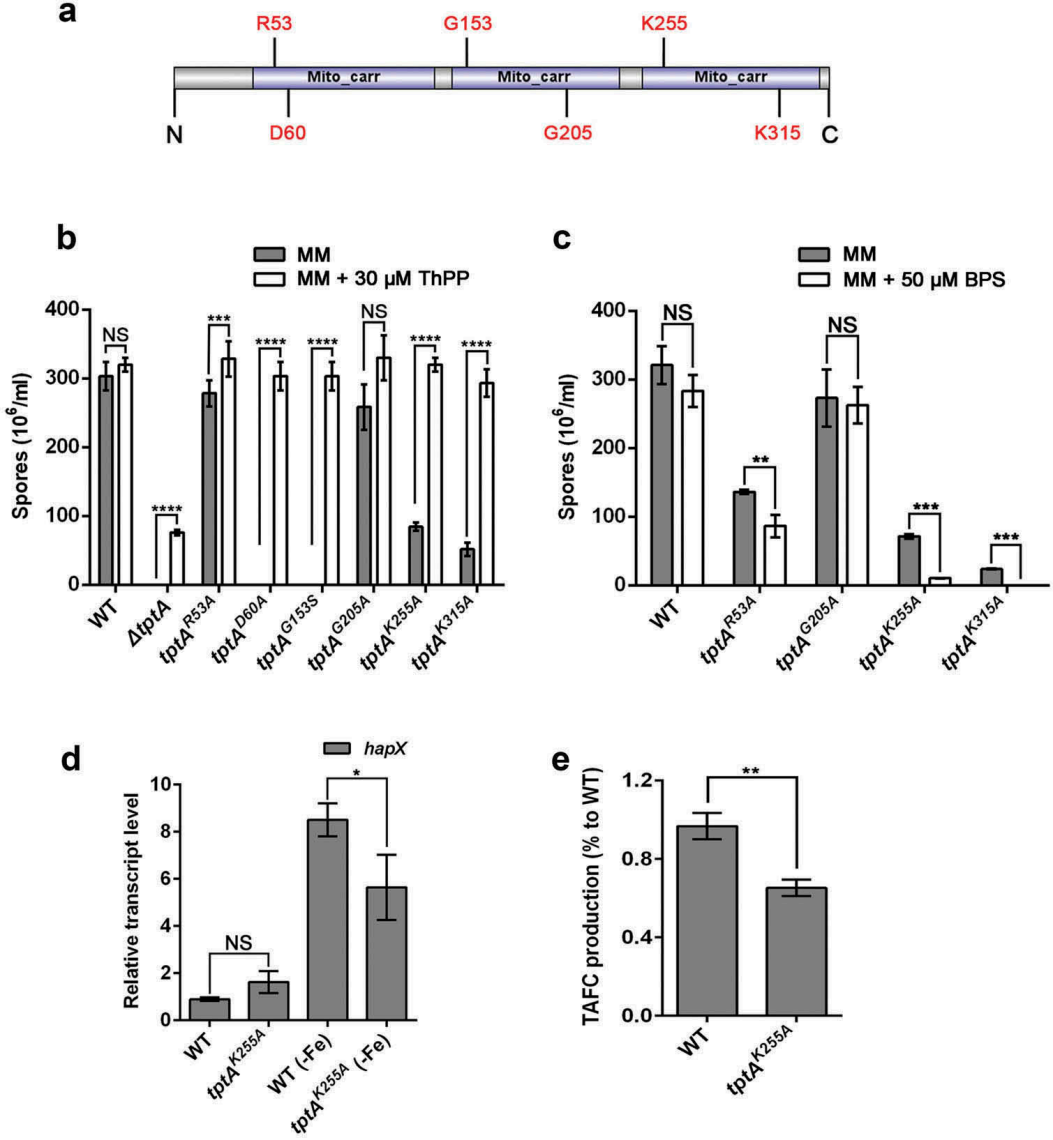
Conserved residues related to thiamine pyrophosphate transport in TptA are required for adaptation to iron-depleted conditions. (a) Schematic view of the TptA mutation sites. (b) Quantitative total conidial production for the indicated strains on MM in the presence or absence of 30 μM ThPP. (c) Quantitation of total conidial production for the indicated strains on MM with or without 50 μM BPS. 2 × 10^4^ conidia of the indicated strains were cultured at 37°C for 48 h. (d) Relative RNA expression of *hapX* in *tptA^K255A^* and wild-type *A. fumigatus* under MM and MM-Fe broth at 37°C for 24 h. (e) TAFC production in *tptA^K255A^* and wild-type *A. fumigatus* in MM-Fe broth. TAFC production of the wild-type was used as a reference. Data are presented as the means ± SD from three independent replicates. “*”, “**”, “***” or “****” represent significant differences at *P < *0.05, *P < *0.01, *P < *0.001 and *P* < 0.0001, respectively, according to t-test.

A link between mitochondrial ThPP transport and the regulation of iron adaptation has not been previously reported. To confirm that these two biological processes were directly linked, we sought to construct TptA proteins with mutations in key residues required for ThPP transport and evaluated the effects of these mutations on adaptation to low iron conditions. Structural characterization of hMTPPT (SLC25A19), a human mitochondrial ThPP transporter, has shown that the conserved residues Thr^29^, Arg^30^, Ile^33^, Ser^34^, Asp^37^ and Phe^298^ have important roles in substrate (ThPP) recognition/interaction. The positively-charged residues His^82^, His^137^, Lys^231^, and Lys^291^ also play important roles in transport [21]. The hMTPPT clinical mutants G125S and G177A displayed significant inhibition of ThPP uptake and a decrease in MTPPT protein expression [22]. Among the above sites, six residues, including Arg^53^, Asp^60^ (potential roles in ThPP recognition/interaction), Lys^255^, Lys^315^ (potential roles in ThPP transport) Gly^153^ and Gly^205^ (related to the hMTPPT clinical mutants), were found to be conserved between SLC25A19 and TptA (Figure S4). A schematic diagram of TptA showing the location of the above-mentioned residues is shown in Figure 5(a).

To understand the importance of the selected conserved residues in TptA function, we mutated each of these six residues individually and examined the effect of each mutation on ThPP transport and low iron adaptation. Colonies of *tptA*^D60A^ and *tptA*^G153S^ exhibited severe growth and conidiation defects on MM without ThPP, suggesting that residues Asp^60^ and Lys^153^ are essential for ThPP transport. In contrast, the *tptA*^R53A^, *tptA*^K255A^ and *tptA*^K315A^ mutants exhibited only mild growth or conidiation defects on MM without ThPP. The *tptA*^G205A^ mutant was indistinguishable from wild-type *A. fumigatus* on MM without ThPP. The addition of 30 μM ThPP completely restored the growth of all five site directed mutants that showed growth defects on MM (*tptA*^D60A^, *tptA*^G153S^, *tptA*^R53A^, *tptA*^K255A^ and *tptA*^K315A^) (Figure 5(b) and Figure S5(a) and (c)). In contrast, only partial restoration was seen in the *ΔtptA* mutant under the same conditions.

Next, we examined the effect of individual mutations on adaptation to low iron conditions. Mutants *tptA*^R53A^, *tptA*^K255A^ and *tptA*^K315A^ showed decreased sporulation when grown on MM plus BPS as compared to MM alone, suggesting these residues are required for fungal low iron adaptation (Figure 5(c) and Figure S5(b) and (d)). In contrast, mutant *tptA*^G205A^ did not exhibit a BPS-sensitive phenotype. The induction of *hapX* expression and TAFC production in the *tptA*^K255A^ mutant was significantly reduced compared to that of wild-type *A. fumigatus* under iron depleted conditions, similar to that seen in the *ΔtptA* mutant (Figure 5(d) and (e)). The *tptA*^D60A^ and *tptA*^G153S^ mutants were not tested for their ability to grow in the presence of BPS, as they exhibited a severe growth defects in the absence of ThPP. These results indicated that the TptA residues required for ThPP uptake are also involved in low iron adaptation, suggesting a direct link between these two pathways.

### The function of TptA in low iron adaptation depends on carbon sources

In *S. cerevisiae, Δtpc1* cells exhibit ThPP auxotrophy when grown in the presence of glucose or galactose, but not when grown on non-fermentative carbon sources such as glycerol, ethanol or acetate [16]. In contrast, TptA is required for the utilization of various carbon sources in *A. fumigatus*. When grown on MM (1% glucose), *ΔtptA* exhibited strict auxotrophy for ThPP. In comparison, slight growth was observed when grown on GMM (1% glycerol) in the absence of ThPP. Moreover, slight growth of the *ΔtptA* mutant was also seen with 2 % potassium acetate, 0.4% acetic acid or 0.4% ethanol as sole carbon sources in the absence of ThPP (Figure S6).

**Figure 6.**
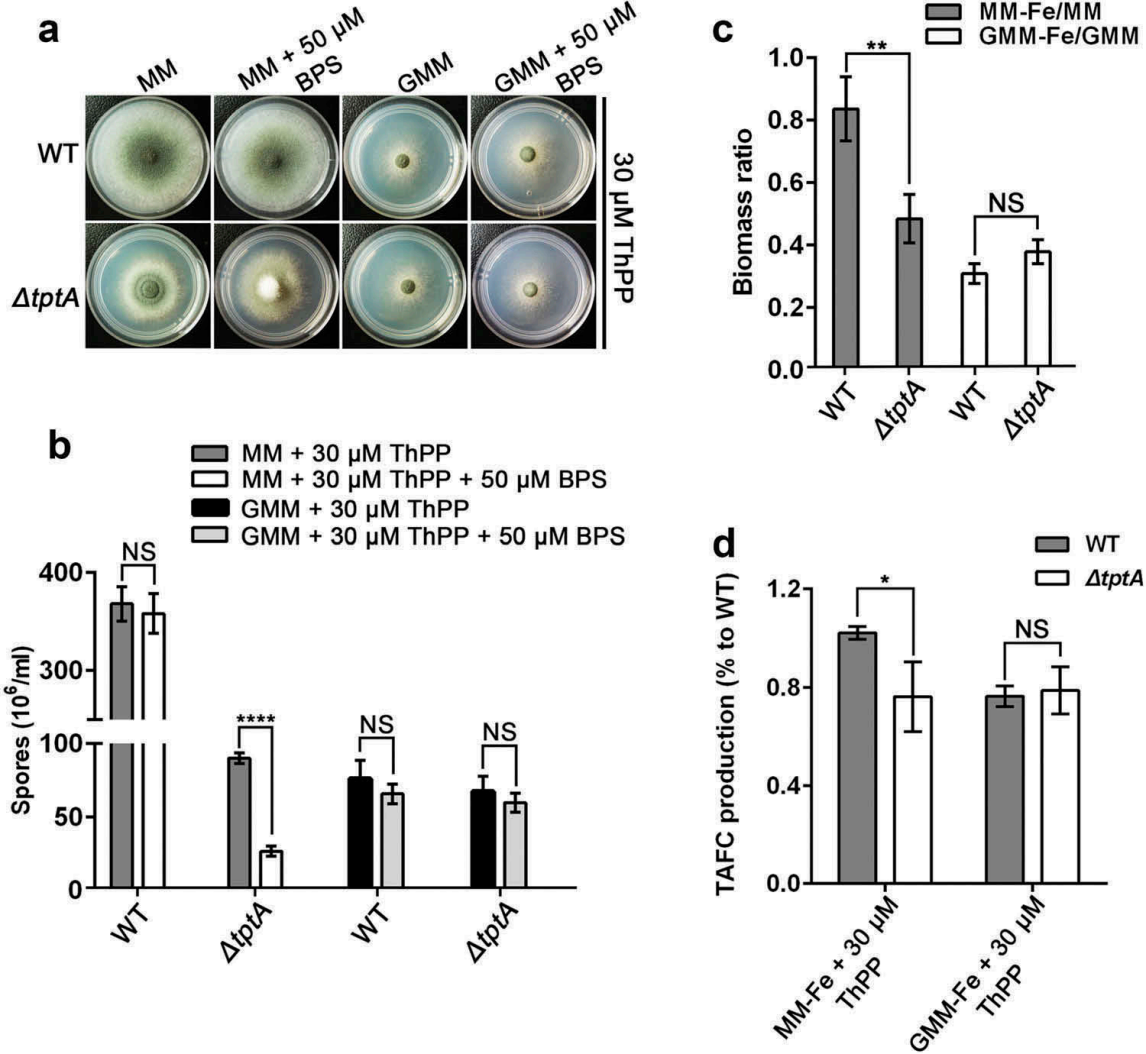
The function of TptA on low iron adaptation depends on carbon sources. (a) Colony morphology of WT and *ΔtptA* mutant grown on GMM (1% glycerol) or MM (1% glucose) with or without 50 μM BPS in the presence of 30 μM ThPP at 37°C for 48 h. (b) Quantitative total conidia production for the strains shown in panel (a). (c) The ratio of biomass production for indicated strains. 1 × 10^8^ conidia of wild-type *A. fumigatus* and *ΔtptA* mutant were inoculated into 100 ml of MM, MM-Fe, GMM or GMM-Fe broth medium in the presence of 30 μM ThPP and cultured at 37°C for 24 h. The biomass was then weighed after freeze drying. (d) Quantification of TAFC production form wild-type *A. fumigatus* and *ΔtptA* mutant on MM-Fe and GMM-Fe broth medium in the presence of 30 μM ThPP. TAFC production of wild-type *A. fumigatus* on MM-Fe was used as a reference. Data are presented as the means ± SD from three independent replicates. “*”, “**” or “****” represents significant differences at *P < *0.05, *P < *0.01 and *P* < 0.0001, respectively, according to t-test.

We further tested BPS sensitivity of the *ΔtptA* mutant on different carbon sources. Consistent with the above observations, the *ΔtptA* mutant showed a dramatically decrease in conidiation, biomass and TAFC production when grown on MM in the presence of 30 μM ThPP (Figure 6(a–d)). In comparison, the *ΔtptA* mutant was indistinguishable from wild-type *A. fumigatus* in terms of conidiation and biomass when grown on GMM in the presence of 30 μM ThPP (Figure 6(a–c)). There was no difference in TAFC production between the *ΔtptA* mutant and wild-type *A. fumigatus* when glycerol was used as a sole carbon source (Figure 6(d)). Collectively, the results suggest that *tptA* was required for the utilization of various carbon sources in *A. fumigatus*. However, the function of TptA in low iron adaptation is dependent on carbon sources.

### TptA deficiency attenuates the virulence of *A. fumigatus* in a non-neutropenic murine model of invasive aspergillosis

**Figure 7.**
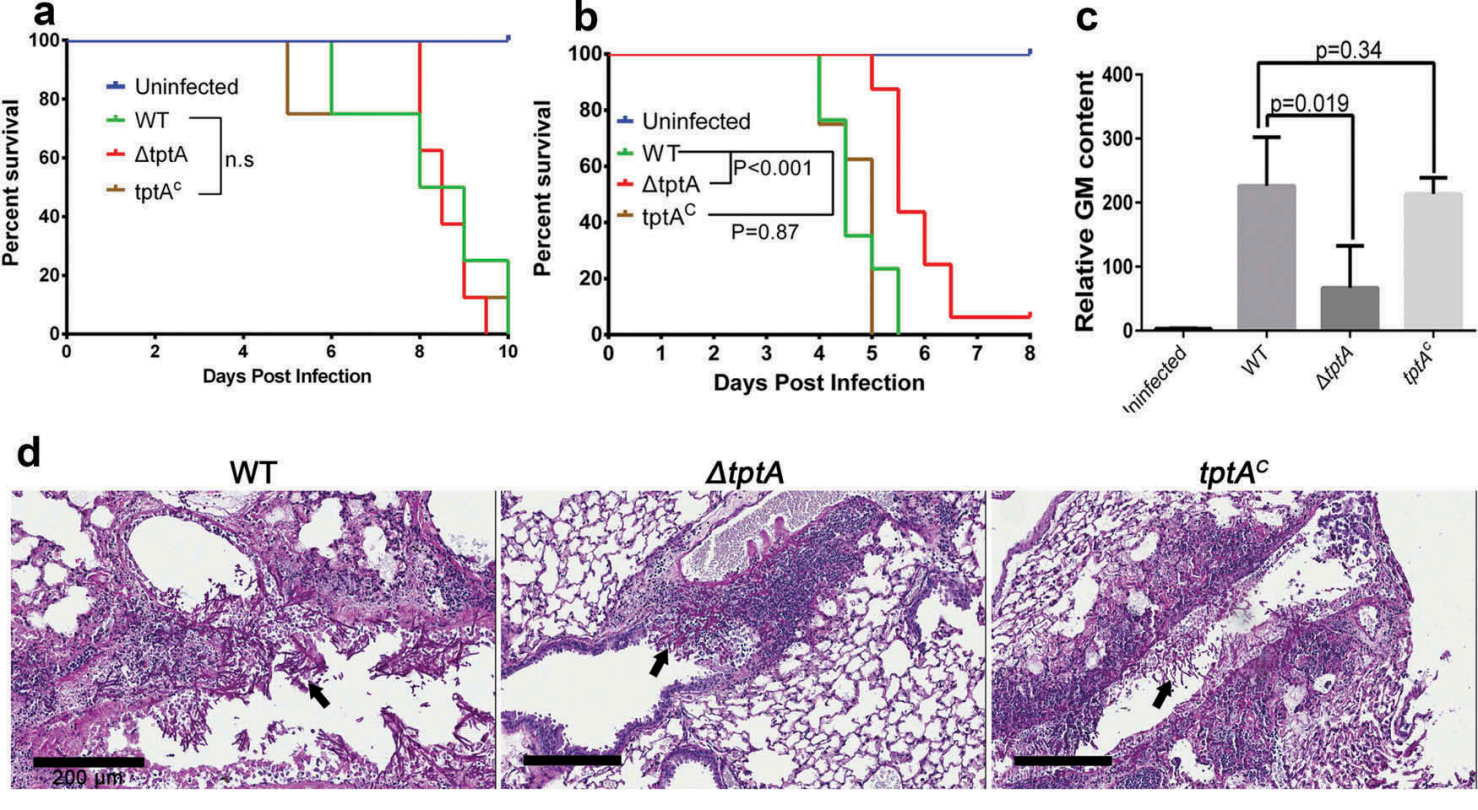
TptA deficiency attenuates virulence of *A. fumigatus* in a murine model of invasive aspergillosis. (a) Survival of highly immunosuppressed mice treated with cyclophosphamide and cortisone acetate and infected with the indicated *A. fumigatus* strains. (b) Survival of cortisone acetate-treated mice infected with the indicated *A. fumigatus* strains. Data are the combined from two independent experiments for a total of 16 mice per strain (n = 16). Statistical differences for mouse survival were calculated using the Log-Rank (Mantel-Cox) test. (c) Pulmonary fungal burden in cortisone acetate-treated mice infected with the indicated strains, as measured by determination of pulmonary galactomannan content (n = 8). Values are medians plus interquartile range (error bars). Statistical differences were calculated using t test. (d) Pulmonary histopathology sections from cortisone acetate-treated mice infected with the indicated strains and stained with PAS for visualization of fungal elements (black arrows). Bar = 200 μM.

To determine whether *tptA* plays a role in fungal pathogenesis, we compared the virulence of the *ΔtptA* strain to that of the complemented *tptA^c^* strain and wild-type *A. fumigatus* in two different mouse models of pulmonary invasive aspergillosis. The models include (i) a leukopenic mouse model with induced immunosuppression from both cortisone acetate and cyclophosphamide, and (ii) a non-leukopenic model with immunosuppression from cortisone acetate [23,24]. No statistically significant difference in the mortality of leukopenic mice infected with wild-type *A. fumigatus, ΔtptA* and *tptA^c^* strains was observed (Figure 7(a)). In contrast, non-leukopenic, cortisone-acetate treated mice infected with the *ΔtptA* mutant survived significantly longer than mice infected with either wild-type *A. fumigatus* or the *tptA*-complemented strain *tptA^c^* (P < 0.001), although more than 90% mortality of *ΔtptA* infected was observed in this model. No statistically significant differences in survival were observed between mice infected with wild-type *A. fumigatus* and the *tptA^c^* mutant (Figure 7(b)).

Consistent with increased survival of mice infected with the *ΔtptA* strain in the cortisone acetate mouse model, mice infected with the *ΔtptA* strain were also found to have significantly reduced pulmonary galactomannan content after three days of infection, when compared with mice infected with wild-type *A. fumigatus* and *tptA^c^* (Figure 7(c)). Moreover, histopathological examination confirmed that infection with the *ΔtptA* mutant strain produced fewer and smaller fungal lesions than the wild-type *A. fumigatus* and *tptA^c^* strain (Figure 7(d)). Thus, *tptA* is required for full virulence of *A. fumigatus*.

## Discussion

Adaptation to iron starvation is crucial for the virulence of pathogenic fungi [2]. Much of what is known about the low iron adaptation mechanisms in fungi was first studied in *S. cerevisiae*. However, pathogenic fungi utilize distinct approaches to acquire iron [25]. T-DNA insertional mutagenesis is an effective forward genetics method to identify novel genes involved in specific processes, including in *A. fumigatus* [26]. Here, we characterized a mutant (T421), in which the integrated T-DNA disrupted *tptA*, a homologue of *tpc1*, which mediates mitochondrial uptake of the essential cofactor ThPP in *S. cerevisiae* [16]. Thiamine, in its active form ThPP, is an essential enzyme cofactor required for the viability of almost all organisms due to its involvement in critical metabolic reactions [27]. Most bacteria, as well as fungi and plants, can produce thiamine *de novo*, but mammals depend solely on dietary uptake, making the involved enzymes highly attractive targets for the design of new selective antifungal compounds. Recently, the thiamine biosynthetic pathway in *A. fumigatus* was analyzed. Loss of *thiB* (Afu2g08970, encoding a thiamine-phosphate diphosphorylase and hydroxyethylthiazole kinase) led to thiamine auxotrophy and attenuated virulence in both pulmonary models (neutropenic and non-neutropenic) of infection and in disseminated infection [28].

ThPP is an essential coenzyme for acetolactate synthase (ALS), pyruvate dehydrogenase (PDH) and oxoglutarate dehydrogenase (OGDH), found in mitochondria [16]. PDH bridges the glycolysis and citric acid cycles *via* acetyl-CoA, ODGH catalyzes a rate-limiting step of the citric acid cycle, and ALS catalyzes the first step in the synthesis of branched-chain amino acids (valine, leucine, and isoleucine). Since ThPP is produced in the cytosol by thiamine pyrophosphokines, the primary function of Tpc1 is most likely to catalyze the uptake of ThPP into mitochondria, where it is required for the activity of ALS, PDH and OGDH [29]. To date, the yeast ThPP carrier (Tpc1p), the human Tpc and the *Drosophila melanogaster* ThPP carrier have been identified as being responsible for mitochondrial transport of ThPP and ThMP [16,30,31]. Homologues of Tpc1 in *Aspergillus* have not yet been functionally characterized. Our results indicated that TptA in *A. fumigatus* fulfills similar cellular roles to Tpc1. Firstly, TptA contains conserved mitochondrial carrier motifs and is located in the mitochondria. Secondly, the addition of ThPP, but not other cofactors, partly restored growth defects in the *ΔtptA* mutant. Moreover, Tpc1 fully restored ThPP auxotrophy in the *ΔtptA* mutant. However, some functional differences between yeast and *A. fumigatus* ThPP carrier proteins were also found. Firstly, in *S. cerevisiae, Δtpc1* cells exhibited thiamine auxotrophy on synthetic MM supplemented with glucose or galactose, but not with the non-fermentative carbon source glycerol, suggesting that ThPP is imported into the mitochondria by a different transporter under these conditions [16]. In contrast, *tptA* is required for the utilization of various carbon sources (including glucose and glycerol). However, it seems that *tptA* plays a more important role in glucose utilization and low iron adaptation than it does in glycerol utilization. Secondly, growth defects in the *Δtpc1* mutant were restored by the simultaneous addition of valine and isoleucine along with the fermentative carbon source glucose, suggesting that ThPP is mainly required in the mitochondria for synthesis of branched chain amino acids [16]. However, the simultaneous addition of valine, isoleucine, and leucine did not restore growth of the *ΔtptA* mutant on glucose, suggesting that *tptA* may play additional essential roles, rather than simply branched chain amino acid synthesis in *A. fumigatus*.

The function of TptA in low iron adaptation is partly due to the regulation of *hapX* expression and subsequent TAFC production. HapX is a bZIP transcription factor that has been shown to mediate adaptation to both iron starvation and iron excess via interaction with the CCAAT binding complex (CBC) [32]. As TptA mediates mitochondrial ThPP import, it appears that *tptA* indirectly regulates *hapX* expression. Considering that TptA is involved in energy metabolism in the cell, its absence may cause an effect on the expression of genes involved in different metabolic pathways, which may include *hapX* mediated iron adaptation. It has been reported that increased heme synthesis can activate CCAAT-binding to the Hap complex and to genes required for the tricarboxylic acid (TCA) cycle, electron transport chain and oxidative phosphorylation in yeast [33]. However, in *A. fumigatus*, it has been reported that *hapX* negatively regulates TCA expression and heme synthesis under iron limited conditions [14]. Whether or how heme or TCA intermediates are able to exert feedback regulation of CBA and *hapX* expression requires further exploration. On the other hand, overexpression of *hapX* did not completely restore the BPS-sensitive phenotype in the *ΔtptA* mutant, suggesting an additional function of *tptA* apart from *hapX* regulation in low iron adaptation.

Virulence attenuation in *A. fumigatus* caused by the loss of *tptA* may reflect the role of ThPP in mitochondria, such as in the TCA cycle and branched chain amino acid biosynthetic pathway, which have been reported to be crucial for both pulmonary and systemic aspergillosis [34]. Additionally, or alternatively, virulence attenuation may be due to the role of TptA in low iron adaptation in the host. This hypothesis was highlighted by the evidence that the *ΔtptA* mutant had attenuated virulence only in the cortisone-acetate model but not in the leukopenic mouse model. In the cortisone-acetate model, the presence of neutrophils and monocytes may increase extracellular iron starvation or impose iron starvation by internalization. Interestingly, the *ΔhapX* mutant also appeared to be slightly more virulent in the leukopenic mouse model compared to the cortisone-acetate model [14]. The above results suggest that genes required for low iron adaptation, such as *hapX* and *tptA*, play a more important role in fungal virulence. Taken together, this study established a link between the mitochondrial ThPP transporter TptA and fungal low iron adaptation using a forward genetics method. TptA was involved in not only ThPP transport into the mitochondria, but also the regulation of *hapX* expression, TAFC production and fungal virulence.

## Materials and methods

### Strains, media and culture conditions

A list of *A. fumigatus* strains used in this study is provided in Table S1. *A. fumigatus* strains were grown on minimal medium (MM) containing 1% (w/v) glucose, 70 mM NaNO_3_ and 18 μM FeSO_4_. Alternatively, YAG medium (consisting of 0.5% yeast extract, 2% glucose, trace elements) was used as a nutrient source. For iron starvation, iron in MM was omitted. To increase iron starvation, the iron-specific chelator bathophenanthroline disulfonate (BPS) was added to MM. Supplementation with iron (FeCl_3_) and/or ThPP was carried out as described in the figure legends. To perform the carbon sources assay, 1% glucose in the MM media was replaced with 2% potassium acetate, 1% glycerol (GMM), 0.4% acetic acid or 0.4% ethanol.

### Plate assays

To analyze the mutant phenotypes, two microliters of conidia from a stock suspension (1 × 10^7^ conidia/ml) of the indicated strains was spotted onto the relevant media. All plates were incubated at 37°C for 48 h except where indicated. The colonies were observed and imaged and the total conidial production of each colony was harvested in 1 ml 0.02% Tween 80 and counted using a hemocytometer.

### Construction and screening of the T-DNA random insertion mutant library of *A. fumigatus*

*Agrobacterium*-mediated transformation was performed, as previously described [35]. In brief, conidia of *A. fumigatus* A293 and *A. tumefaciens* strain EHA105 were co-cultivated at a ratio of 1:10 (conidia to bacteria) on induction medium supplemented with 200 μM acetosyringone, a phenolic compound that induces transfer-DNA (T-DNA) to enter the recipient strain. After co-cultivation for 48 h at 24°C, YAG medium supplemented with hygromycin (300 μg/ml) and cefotaxime (200 μg/ml) was used to select transformants. Transformants possessing hygromycin resistance were inoculated onto MM containing 200 μM BPS at 37°C for 48 h. The transformants shown to be more sensitive to iron starvation conditions compared to *A. fumigatus* A293 were isolated for further analysis.

### Cloning of unknown flanking sequences

To determine the T-DNA insertion sites, thermal asymmetric interlaced (TAIL)-PCR was performed as previously described [17]. TAIL-PCR is commonly composed of three nested amplifications. The primers used in each amplification reaction consisted of left or right border primers, corresponding to the border sequence of the T-DNA, and an AD primer. All TAIL-2 and TAIL-3 products were sequenced and the resulting sequences were then used as queries in BLAST analyses against the *A. fumigatus database*. Primers used in this study are shown in Table S 2.

### Construction of *tptA* gene deletion and complemented strains

To construct a *tptA* knockout strain, fusion PCR was used as previously described [36]. In brief, approximately 1 kb sections of the regions flanking the *tptA* gene were amplified using primers tptA P1/P3 and tptA P4/P6. The selective marker *pyr4* (approximately 2 kb in length) from the plasmid pAL5 was amplified using primers Pyr4 F/R. Next, the three previously mentioned PCR products were used as a template to generate the *tptA* deletion cassette using primers tptA P2/P5 and then transformed into the *A. fumigatus* strain A1160 [36]. Transformants were verified by diagnostic PCR using primers tptA SF/SR, tptA P1/Pyr4 down and Pyr4 up/tptA P6 and Southern blotting.

For the construction of the *ΔtptA* complemented strain *tptA^C^*, a PCR-generated DNA fragment including the *tptA* open reading frame (ORF) plus approximately 1 kb upstream of the ATG start codon and 1 kb downstream of the stop codon, was obtained using primers tptA-up-XbaI and tptA-down-HindIII. Subsequently, this fragment was cloned into *Xba*I and *Hind*III digested pAN7-1, which contains the hygromycin B resistance gene *hph*, to generate the *tptA* complementation plasmid, pTptA-com-hph. The plasmid pTptA-com-hph was then transformed into the *tptA* deletion strain and transformants were selected on YAG media supplemented with 200 μg/ml hygromycin.

To complement the T421 mutant with the wild-type *A. fumigatus tptA*, fusion PCR was used as previously described. In brief, an approximately 3 kb fragment, including the native promoter, the 5ʹ UTR, *tptA* ORF and the 3ʹ UTR, was amplified using primers tptA P1/tptA-com P6. The selective marker *ptrA* from the plasmid pCH008 was amplified with primers PtrA F/R. The two fragments were then fused and subcloned into pEASY-Blunt zero (TransGen Biotech) to obtain the plasmid pTptA-com-ptrA. The plasmid pTptA-com-ptrA was then transformed into the T421 mutant and transformants were selected on MM supplemented with 0.1 μg/ml pyrithiamine to obtain the *tptA::T421* strain.

### Overexpression of *Sctpc1* in T421 and *ΔtptA* mutants

To overexpress *S. cerevisiae tpc1* (*Sctpc1*) in the *tptA* mutant background, the hygromycin B resistance gene *hph* was amplified using primers hph-up-SpeI and hph-down-SpeI and then cloned into the *Spe*I site of pBARGPE to generate pBARGPE-hph. The *Sctpc1* ORF was amplified from *S. cerevisiae* S288c genomic DNA with the primers OE::Sctpc1-up-EcoRI and OE::Sctpc1-down-EcoRI and then subcloned into the *EcoR*I site of pBARGPE-*hph* to generate a *tpc1* overexpression plasmid, pOEtpc1-hph. The plasmid pOEtpc1-hph was transformed into the *tptA* deficient strain to obtain the *OESctpc1::tptA* strain. To overexpress *Sctpc1* in the T421 mutant background, an approximately 1.8 kb fragment including the *tpc1* ORF and constitutive promoter *gpdA* from the plasmid pOEtpc1-hph was amplified with primers gpd F and Sctpc1 R. The aforementioned selective marker *ptrA* was amplified with Sctpc1-PtrA F/PtrA R. The two fragments were then fused using primers gpd F/PtrA R and the resultant PCR product was transformed into the T421 mutant to obtain the *OESctpc1::T421* strain.

### Creation of *hapX* deletion and overexpression strains

1 kb sections of the flanking regions of the *hapX* gene were amplified with the primer pairs hapX P1/P3 and hapX P4/P6. The selective marker *hph* from pAN7-1 was amplified with primers Hph F/R. The same approach to that described previously was taken to create a *hapX* deletion cassette with primers hapX P2/P5 and then the resulting PCR product was transformed into the *ΔtptA* mutant background and parental wild-type *A. fumigatus* to generate the *ΔhapXΔtptA* strain and *ΔhapX* strain, respectively.

The *hapX* ORF from *A. fumigatus* A1161 genomic DNA was amplified with the primers OEhapX-up-ClaI and OEhapX-down-ClaI. The generated fragment was then subcloned into the *Cla*I site of pBARGPE-hph to obtain pOEhapX-hph. The plasmid pOEhapX-hph was transformed into the *tptA* deletion strain and parental wild-type to generate the strains *OEhapX::ΔtptA* and *OEhapX*, respectively.

### Construction of TptA::GFP fusion strain

To create a TptA::GFP cassette, a GFP + pyrG fragment was amplified from the plasmid pFNO3 using primer pairs GFP + PyrG F/R. The same approach to that described previously [36] was used to construct the TptA::GFP fusion cassette. In brief, a 1 kb fragment immediately upstream of the *tptA* stop codon and a 1 kb fragment immediately downstream of the *tptA* stop codon were amplified using primer pairs TptA-GFP P1/P3 and TptA-GFP P4/P6, respectively. The TptA::GFP fusion PCR cassette (amplified using primer pairs TptA-GFP P2/P5) was transformed into strain A1160 and homologous recombination was verified by PCR using primers TptA-GFP P1/GFP + Pyr4 R and GFP + Pyr4 F/TptA-GFP P6.

### Construction of TptA point mutation strains

For site-directed mutagenesis, complementary primers, approximately 20 bp in length that included the desired mutation in the center position, were designed and synthesized. The plasmid pTptA-com-hph, harboring the wild-type *A. fumigatus tptA* gene, was used as a template. Two fragments containing the desired mutation were amplified using tptA-up-XbaI or tptA-down-HindIII with their respective primer pairs and the resulting PCR products were treated with *Dpn*I to remove the template. The two fragments were then cloned into *Xba*I and *Hind*III digested pAN7-1 using the ClonExpressII One Step Cloning Kit (Vazyme). The related plasmids were transformed into the *ΔtptA* mutant to obtain the strains referred to as *tptA^R53A^, tptA^D60A^, tptA^G153S^, tptA^G205A^, tptA^K255A^ and tptA^K315A^*.

### Identification and quantification of extracellular siderophore (TAFC)

To identify and quantify siderophore (TAFC) production, strains were cultured under iron depleted conditions (MM-Fe) for 24 h. The supernatants were then filtered and excess FeCl_3_ was added (to a final concentration of 1.5 mM) to convert desferri-siderophores to the ferri-forms. Ferric-TAFC was absorbed with Amberlite XAD-16 resin (CWG) equilibrated with 50 mM potassium phosphate buffer (pH 7.5). The resin was subsequently washed with 10 ml of phosphate buffer and TAFC was eluted with 5 ml of methanol [37]. Methanol was removed by evaporation, the TAFC was resuspended in 500 μl of double-distilled water and 30 μl was analyzed by reversed-phase HPLC. Isolated ferric-TAFC was quantified photometrically at 435 nm [38].

### Southern blotting

Genomic DNA from the appropriate strains was digested with *Xho*I, separated by electrophoresis at 80 V for 1.5 h and transferred to a nylon membrane. A 0.7 kb fragment amplified with the primers tptA probeF and tptA probeR was used as a probe. Labeling and visualization were performed using a DIG DNA labeling and detection kit (Roche Applied Science), according to the manufacturer’s instructions.

### RNA extraction for qRT-PCR

3 × 10^7^ conidia of the parental wild-type were inoculated into 100 ml MM or MM-Fe liquid media and incubated at 37°C for 24 h. To compensate for the reduced growth rate and to yield the same biomass, 1 × 10^8^ conidia of *ΔtptA* and *tptA*^K255A^ mutants were cultured under the same conditions. Total RNA was isolated from mycelia with TRIzol (Roche) as described in the manufacturer’s instructions. Genomic DNA digestion and cDNA synthesis were performed using HiScript II Q RT SuperMix for qPCR kit (Vazyme), according to the manufacturer’s instructions. The qRT-PCR was performed using an ABI One-step fast thermocycler (Applied Biosystems) with AceQ qPCR SYBR Green Master Mix (Vazyme). *Tubulin* was used as an internal control for qRT-PCR. All qRT-PCR primers are shown in Table S2 in the supplementary data.

### Fluorescence microscopy

To localize TptA::GFP, fresh conidia were inoculated onto sterile glass coverslips overlaid with 1 ml of liquid MM for 14 h before observation. The coverslips with hyphae were gently washed three times with phosphate buffered saline (PBS). The mitochondrial marker MitoTracker Red (Invitrogen), dissolved in PBS, was added at a final concentration of 50 nM and incubated for 5 min at room temperature [39]. Images were captured using a Zeiss Axio Imager A1 microscope (Zeiss, Jena, Germany) and images were prepared using Adobe Photoshop.

### Virulence assay

Virulence of the *A. fumigatus* strains was tested in two different murine models of invasive pulmonary aspergillosis [40]. In the first model, 5 to 6 week old male BALB/C mice, were immunosuppressed with 500 mg/kg cortisone acetate subcutaneously (Sigma-Aldrich) every other day, starting on day −4 relative to infection and finishing on day +2, for a total of 4 doses. In the second model, mice were immunosuppressed with cortisone acetate, (250 mg/kg subcutaneously) on days −2 and +3 and cyclophosphamide (Western Medical Supply) at 250 mg/kg intraperitoneally on day −2 and 200 mg/kg on day +3. To prevent bacterial infection, enrofloxacin was added to the drinking water (Baytril, Inc.). Mice were infected with an endotracheal injection of 5 × 10^3^ *A. fumigatus* conidia resuspended in PBS. Mice were monitored daily and moribund animals were euthanized. For determination of pulmonary fungal burden, the lungs were harvested at day 3 post-infection and were either homogenized to determine the fungal burden or fixed in formalin for histopathology. The fungal burden in lung homogenates was quantified by determining relative galactomannan content as previously described [41]. For pulmonary histopathology examination, lung sections were stained with periodic acid-Schiff (PAS) and HE. All procedures involving mice were approved by the McGill University Animal Use and Care Committee.

## Supplementary materials

The supplementary material for this article can be accessed here.
