# Supplementary material for: The mitochondrial thiamine pyrophosphate transporter TptA promotes adaptation to low iron conditions and virulence in fungal pathogen Aspergillus fumigatus
Source: Virulence. 2019 Mar 28;10(1):234–47. doi: 10.1080/21505594.2019.1596505 (PMC6527022; doi:10.1080/21505594.2019.1596505)
Supplement: Supplemental Material [file kvir-10-01-1596505-s001.zip › Table S1.docx]

Table S1 *Aspergillus fumigatus* strains used in this study.

| Strains | Genotype | Reference |
| --- | --- | --- |
| A293 | *wild type* | FGSC |
| A1160 | *Δku80, pyrG1* | FGSC |
| WT | *Δku80, A1160::pyrG1* | [[1](#_ENREF_1)] |
| T421 | A293*::T-DNA::hph* | This study |
| *tptA::T421* | A293*::T-DNA::hph, tptA(p)::tptA::ptrA* | This study |
| *OESctpc1::T421* | A293*::T-DNA::hph, gpd(p)::Sctpc1::ptrA* | This study |
| *ΔtptA* | *Δku80, pyrG1, ΔtptA::pyr4* | This study |
| *tptA^C^* | *Δku80, pyrG1, ΔtptA::pyr4, tptA(p)::tptA::hph* | This study |
| *OESctpc1::ΔtptA* | *Δku80, pyrG1, ΔtptA::pyr4, gpd(p)::Sctpc1::hph* | This study |
| TptA::GFP | *Δku80, pyrG1, tptA::GFP::pyrG1* | This study |
| *OEhapX* | *Δku80, A1160::pyrG1, gpd(p)::hapX::hph* | This study |
| *OEhapX::ΔtptA* | *Δku80, pyrG1, ΔtptA::pyr4, gpd(p)::hapX::hph* | This study |
| *ΔhapX* | *Δku80, A1160::pyrG1, ΔhapX::hph* | This study |
| *ΔhapXΔtptA* | *Δku80, pyrG1, ΔtptA::pyr4, ΔhapX::hph* | This study |
| *tptA^R53A^* | *Δku80, pyrG1, ΔtptA::pyr4, tptA(p)::tptA^(R53A)^::hph* | This study |
| *tptA^D60A^* | *Δku80, pyrG1, ΔtptA::pyr4, tptA(p)::tptA^(D60A)^::hph* | This study |
| *tptA^G153S^* | *Δku80, pyrG1, ΔtptA::pyr4, tptA(p)::tptA^(G153S)^::hph* | This study |
| *tptA^G205A^* | *Δku80, pyrG1, ΔtptA::pyr4, tptA(p)::tptA^(G205A)^::hph* | This study |
| *tptA^K255A^* | *Δku80, pyrG1, ΔtptA::pyr4, tptA(p)::tptA^(K255A)^::hph* | This study |
| *tptA^K315A^* | *Δku80, pyrG1, ΔtptA::pyr4, tptA(p)::tptA^(K315A)^::hph* | This study |

1. Jiang H, Shen Y, Liu W, Lu L (2014) Deletion of the putative stretch-activated ion channel Mid1 is hypervirulent in *Aspergillus fumigatus*. Fungal Genet Biol 62: 62-70.
