# Supplementary material for: The mitochondrial thiamine pyrophosphate transporter TptA promotes adaptation to low iron conditions and virulence in fungal pathogen Aspergillus fumigatus
Source: Virulence. 2019 Mar 28;10(1):234–47. doi: 10.1080/21505594.2019.1596505 (PMC6527022; doi:10.1080/21505594.2019.1596505)
Supplement: Supplemental Material [file kvir-10-01-1596505-s001.zip › Table S2.docx]

Table S2 Primers used in this study.

| Primers- | Sequence (5’-3’) | Purpose |
| --- | --- | --- |
| LB1 | GTGTAAAGCCTGGGGTGCCTAATGAGTG | TAIL-PCR |
| LB2 | AGCTAACTCACATTAATTGCGTTGCG |  |
| LB3 | CGGGGAGAGGCGGTTTG |  |
| RB1 | GGCACTGGCCGTCGTTTTACAAC |  |
| RB2 | AACGTCGTGACTGGGAAAACCCT |  |
| RB3 | CCCTTCCCAACAGTTGCGCA |  |
| AD1 | WGTGNAGWANCANAGA |  |
| AD2 | AGWGNAGWANCAWAGG |  |
| AD3 | NGTCGASWGANAWGAA |  |
| AD4 | GTNCGASWCANAWGTT |  |
| tptA P1 | GATTCAGGAGGGTTGGCTTTC | Construct a *tptA* knockout strain |
| tptA P2 | GCTCCATCCCTTGATATCAG |  |
| tptA P3 | CGATTAAGTTGGGTAACGCCAGCATGTGATGTGCGAAGCAA |  |
| tptA P4 | ATAAGTAGCCAGTTCCCGAAAGCCGTGAATGAAAACAACCCACC |  |
| tptA P5 | TGAGAACTCTGACTGCACCT |  |
| tptA P6 | ACTGCCGTTGTTGAACCCTAC |  |
| tptA SF | CCTTAAAAGACCCCGCTGAT |  |
| tptA SR | CGAGTTGTGCGAGAATTTGG |  |
| Pyr4 F | TGGCGTTACCCAACTTAATCG |  |
| Pyr4 R | GCTTTCGGGAACTGGCTACTTAT |  |
| Pyr4 down | GATGCGCTCCCTCCACTTTTG |  |
| Pyr4 up | GACACGGATGGGAGGAAGAGTA |  |
| tptA-up-XbaI | GAGGTAATCCTTCTTTCTAGAGATTCAGGAGGGTTGGCTTTC | Complement *tptA* null mutant |
| tptA-down-HindⅢ | ACGACGGCCAGTGCCAAGCTTACTGCCGTTGTTGAACCCTAC |  |
| hph-up-SpeⅠ | CGGACTAGTGAATTCCCTTGTATCTCTAC |  |
| hph-down-SpeI | CGGACTAGTTCGAGTGGAGATGTGGA |  |
| tptA-com P6 | CATCGGCGGACGATCTCCTAGACTGCCGTTGTTGAACCCTAC | Complement *tptA* in T421 strain |
| PtrA F | CTAGGAGATCGTCCGCCGATG |  |
| PtrA R | GCCTCTTGCATCTTTGTTTGTA |  |
| OESctpc1-up-EcoRI | GGGCTGCAGGAATTCGATATCATGTTCAAAGAGGAGGACTCG | Heterologously overexpress *Sctpc1*  in *tptA* null mutant |
| OESctpc1-down-EcoRI | GGTATCGATAAGCTTGATATCGGAAACTTTAGTACATCCGC |  |
| gpd F | TCCCGGCATCCGCTTACAGAC | Heterologously overexpress *Sctpc1*  in T421 strain |
| Sctpc1 R | TTAGTACATCCGCAAATAATGG |  |
| Sctpc1-PtrA F | CCATTATTTGCGGATGTACTAACTAGGAGATCGTCCGCCGATG |  |
| TptA-GFP P1 | CTGATGAAAGGTTCCTGAGCAC | Create a TptA::GFP cassette |
| TptA-GFP P2 | TTGCTTCGCACATCACATGC |  |
| TptA-GFP P3 | CCAGCGCCTGCACCAGCTCCTTCAGCGACGCGGAAGTCGC |  |
| TptA-GFP P4 | CATCAGTGCCTCCTCTCAGACAGTGACGTGAATGAAAACAACCC |  |
| TptA-GFP P5 | TGAGAACTCTGACTGCACCT |  |
| TptA-GFP P6 | GTAGGGTTCAACAACGGCAGT |  |
| GFP + Pyr4 F | GTGAAGAGCATTGTTTGAGGC |  |
| GFP + Pyr4 R | GATACAGGTCTCGGTCCCTA |  |
| R53A R | TGTACTCACGCCGAGACCAG | Construct site-directed mutants |
| R53A F | CTGGTCTCGGCGTGAGTACA |  |
| D60A R | TCTTGACGACGGCTAGAGGGG |  |
| D60A F | CCCCTCTAGCCGTCGTCAAGA |  |
| G153S R | GCTACAGCGGACGAGACGAA |  |
| G153S F | TTCGTCTCGTCCGCTGTAGC |  |
| G205A R | GCACTGCAGGCGCGGAAGAA |  |
| G205A F | TTCTTCCGCGCCTGCAGTGC |  |
| K255A R | ACCCCGCTCGCGGCCAGAAC |  |
| K255A F | G TTCTGGCCGCGAGCGGGGT |  |
| K315A R | GGTGCGGCCGCGATCAAGCT |  |
| K315A F | AGCTTGATCGCGGCCGCACC |  |
| hapX P1 | TCCAGGACTGATAACCACG | Create *hapX* deletion cassette |
| hapX P2 | AGTAAGTAGTTGCTGTGCG |  |
| hapX P3 | GCCTGTGTGTAGAGATACAAGGGAATTCGATTACGGATGATGAGACT |  |
| hapX P4 | TAAGCGCCCACTCCACATCTCCACTCGATTTATCGCATCTCTGCTTG |  |
| hapX P5 | GAAGTGATGGTTAGTGGTG |  |
| hapX P6 | CTTTTCTGGGGTCTGGTCT |  |
| hapX SF | ACCAAAACCAGGCAGGAAA |  |
| hapX SR | GGCAAATCGGGAAGTGAAA |  |
| Hph F | GAATTCCCTTGTATCTCTACACACAGGC |  |
| Hph R | TCGAGTGGAGATGTGGAGTGGGCGCTTA |  |
| OEhapX-up-ClaI | ACCTTTAATCAAGCTTATCGATCCTACCATTCTCCTCCACCC | Construct *hapX* overexpression cassette |
| OEhapX-down-ClaI | CTCGAGGTCGACGGTATCGATCGAGTCCGTTTGGGTATCAT |  |
| tptA probeF | CATCCCATGTCGGTCCATAG | Synthesis probe for Southern blotting |
| tptA probeR | AGGAACAGGCACAGGGCTTA |  |
| RT-tub F | TTCCGTCCCGACAACTTCGT | RT-qPCR |
| RT-tub R | CACAGCCTTCAGCCTCACG |  |
| RT-hapX F | CCGCACCATCCTTGACTTTAT |  |
| RT-hapX R | CGAGGTTTAGGCAAGGTATGAA |  |
| RT-sreA F | CGATTTCCACTCTTCTAAACCACT |  |
| RT-sreA R | GAGGCTTTCAAGTTCAGACACG |  |
| RT-tptA F | GGGATAACAGGCCTCTGGAAAGG |  |
| RT-tptA R | GCGCCCGAGACGAAAGATTC |  |
|  |  |  |
