# Supplementary figures and images for: The mitochondrial thiamine pyrophosphate transporter TptA promotes adaptation to low iron conditions and virulence in fungal pathogen Aspergillus fumigatus
Source: Virulence. 2019 Mar 28;10(1):234–47. doi: 10.1080/21505594.2019.1596505 (PMC6527022; doi:10.1080/21505594.2019.1596505)

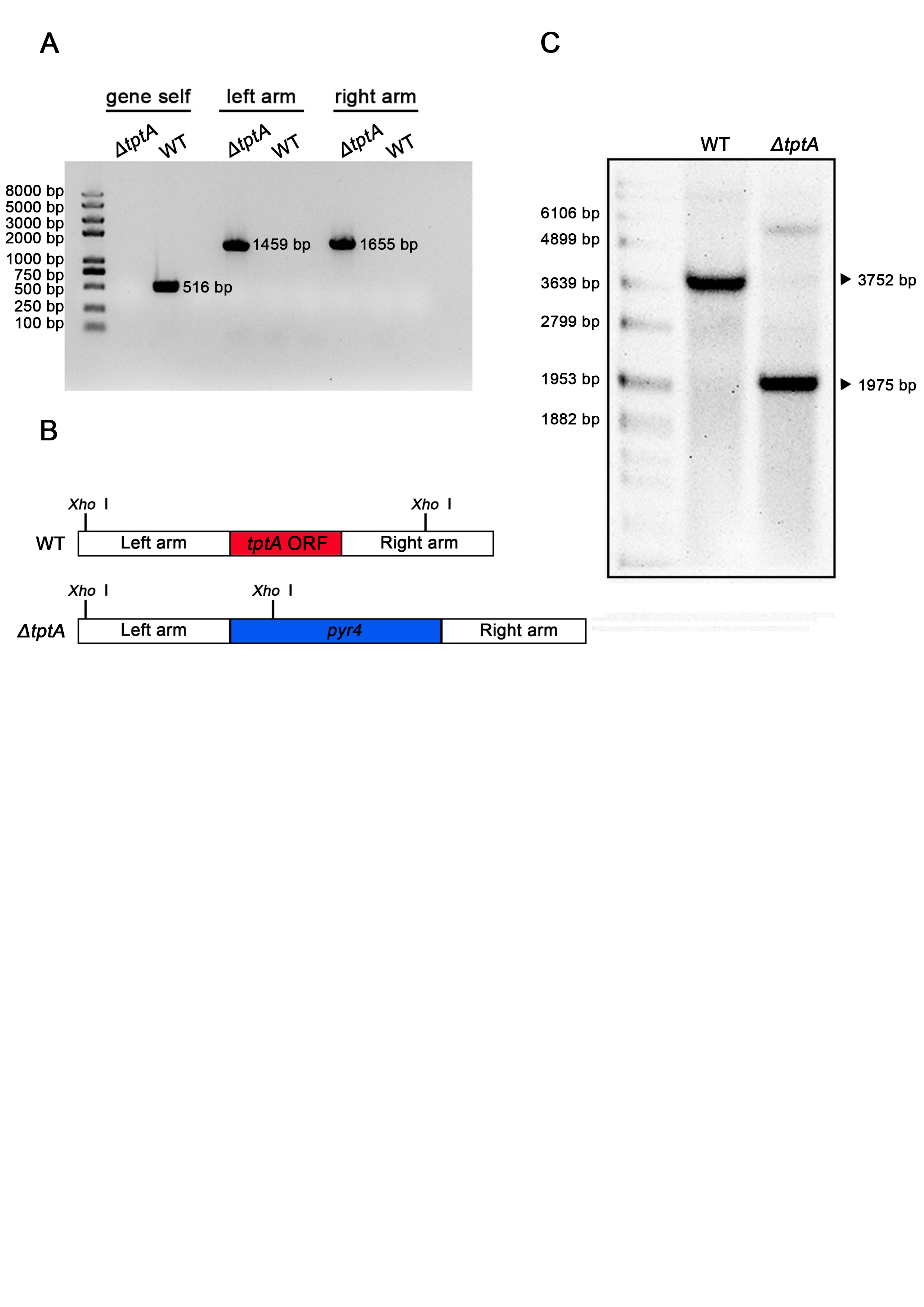

Supplement: Supplemental Material [file kvir-10-01-1596505-s001.zip › FIG S1.jpg]

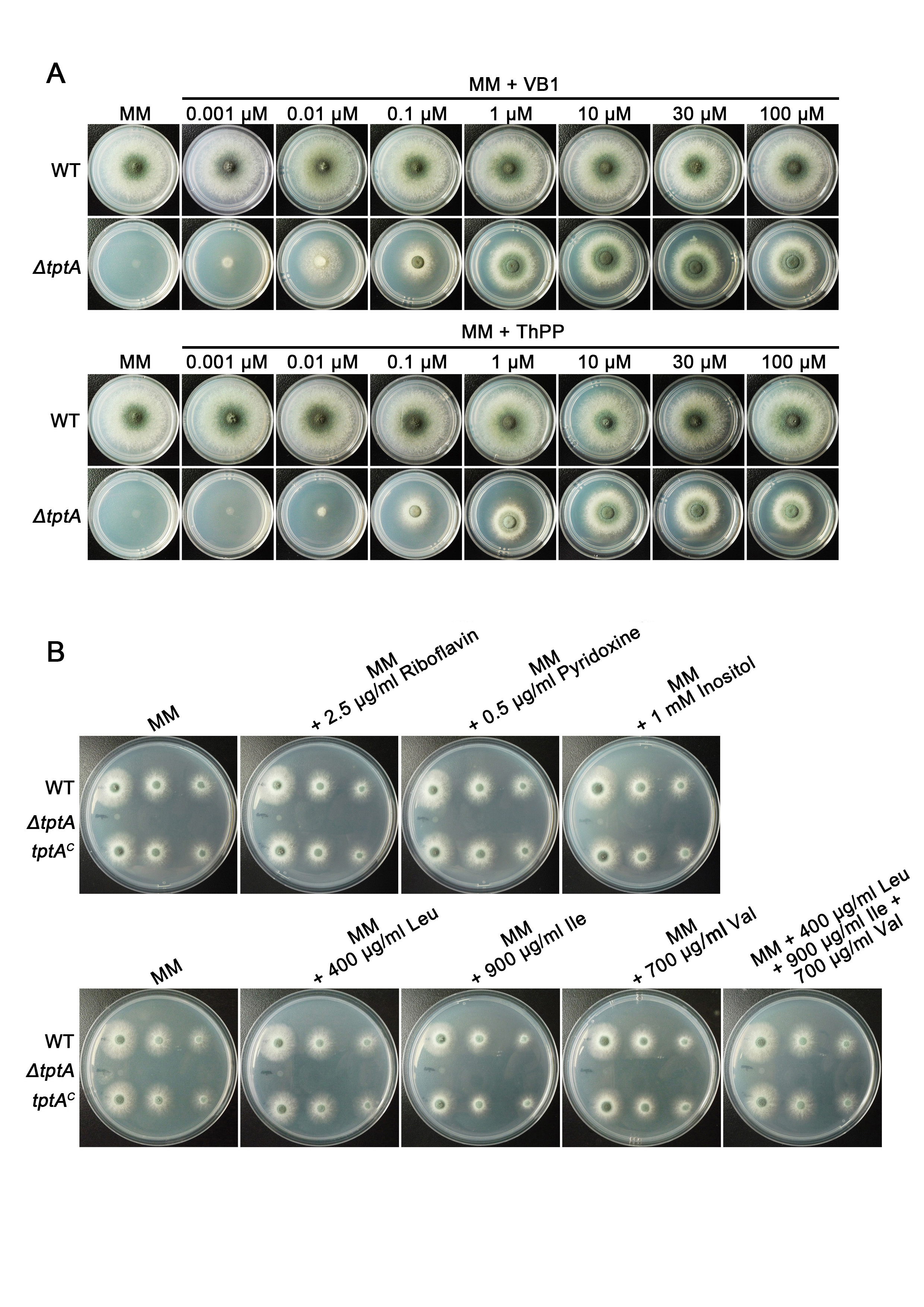

Supplement: Supplemental Material [file kvir-10-01-1596505-s001.zip › FIG S2.jpg]

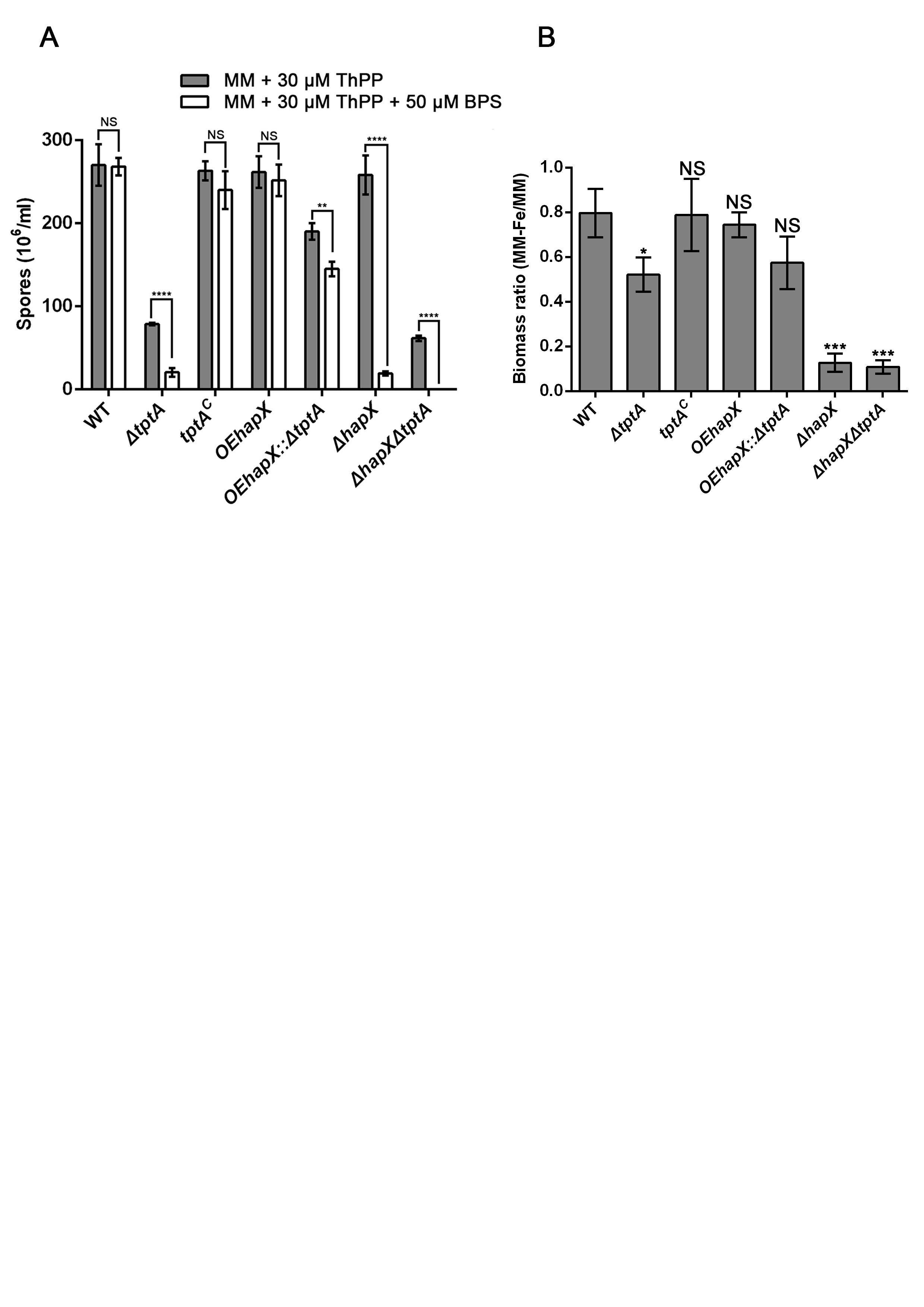

Supplement: Supplemental Material [file kvir-10-01-1596505-s001.zip › FIG S3.jpg]

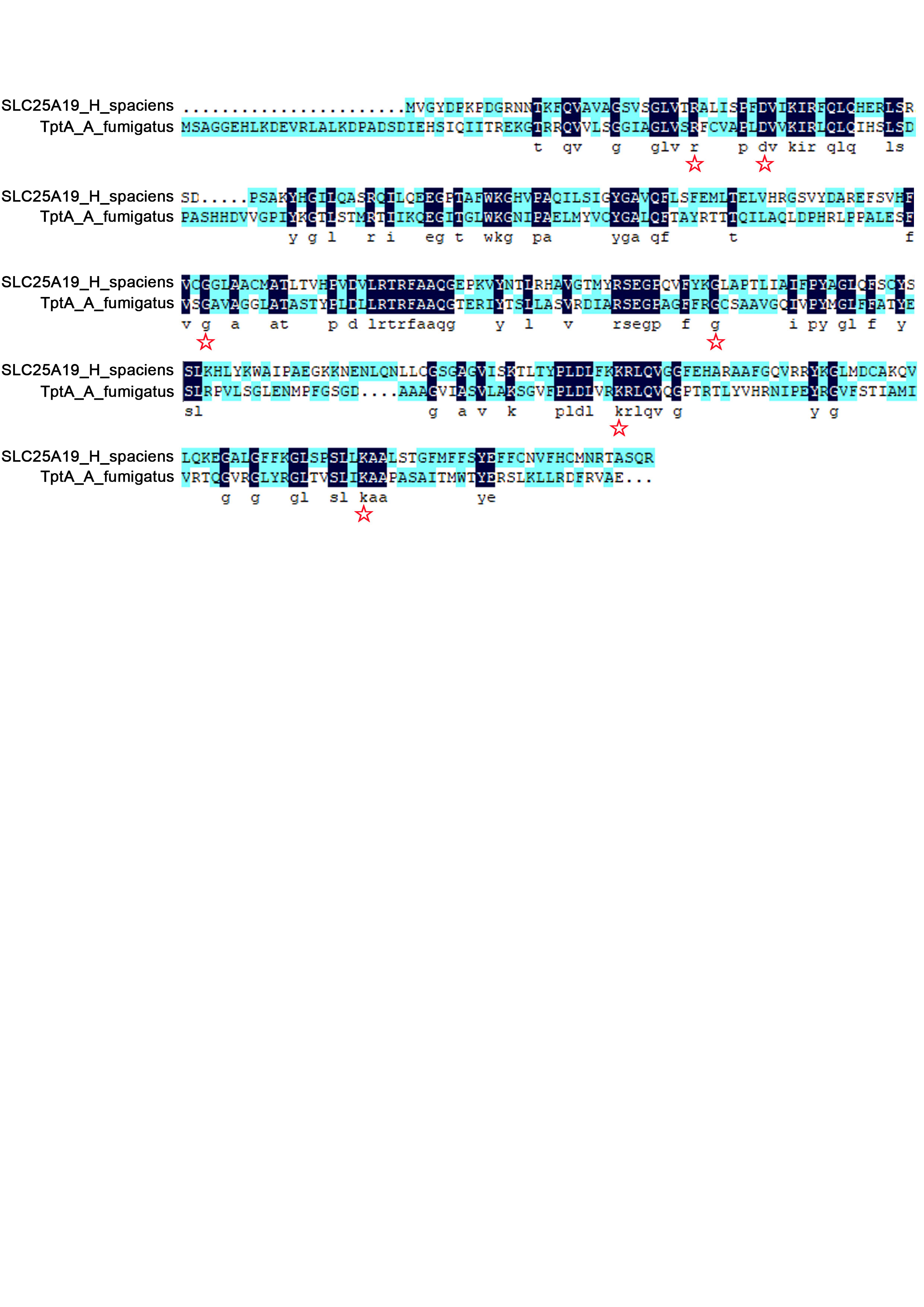

Supplement: Supplemental Material [file kvir-10-01-1596505-s001.zip › FIG S4.jpg]

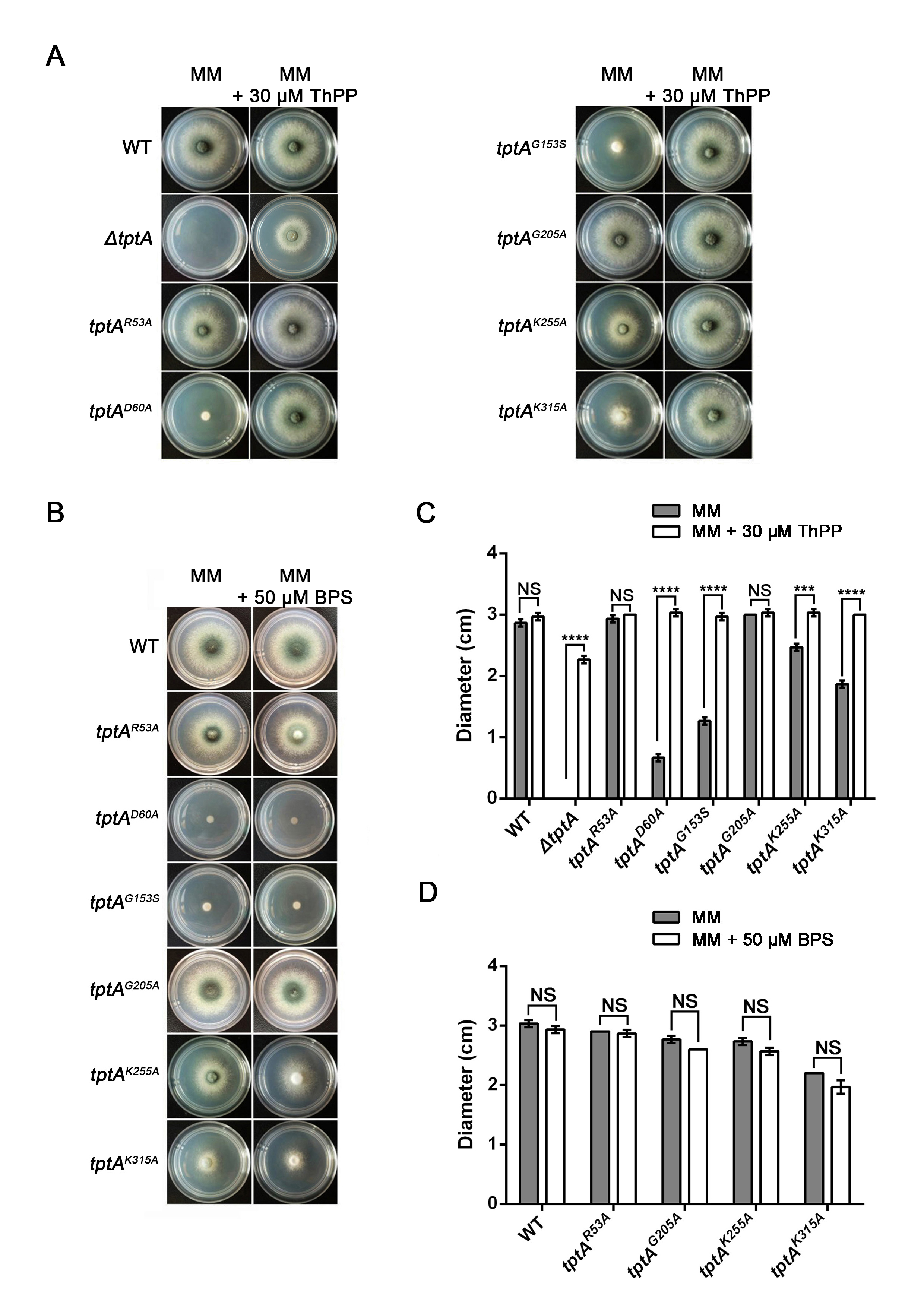

Supplement: Supplemental Material [file kvir-10-01-1596505-s001.zip › FIG S5.jpg]

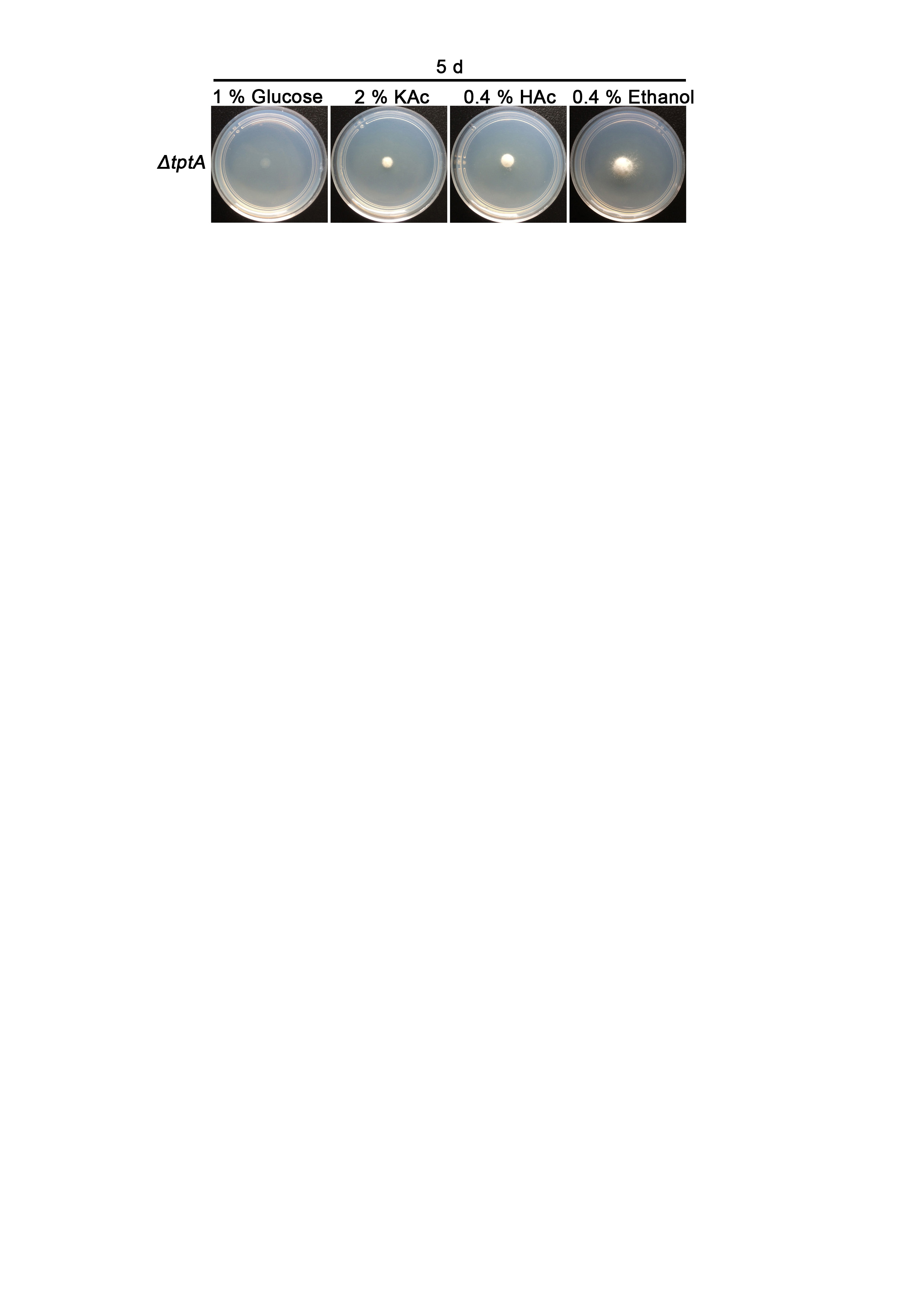

Supplement: Supplemental Material [file kvir-10-01-1596505-s001.zip › FIG S6.jpg]
